# An Abbreviated Protocol for In Vitro Generation of Functional Human Embryonic Stem Cell-Derived Beta-Like Cells

**DOI:** 10.1371/journal.pone.0164457

**Published:** 2016-10-18

**Authors:** Mohammad Massumi, Farzaneh Pourasgari, Amarnadh Nalla, Battsetseg Batchuluun, Kristina Nagy, Eric Neely, Rida Gull, Andras Nagy, Michael B. Wheeler

**Affiliations:** 1 Departments of Medicine and Physiology, Faculty of Medicine, University of Toronto, ON, Canada; 2 Toronto General Hospital Research Institute, University Health Network, Toronto, ON, Canada; 3 Lunenfeld-Tanenbaum Research Institute, Mount Sinai Hospital, Toronto, ON, Canada; 4 Department of Biomedical Sciences, Faculty of Health and Medical Sciences, University of Copenhagen, Copenhagen, Denmark; University of Bremen, GERMANY

## Abstract

The ability to yield glucose-responsive pancreatic beta-cells from human pluripotent stem cells *in vitro* will facilitate the development of the cell replacement therapies for the treatment of Type 1 Diabetes. Here, through the sequential *in vitro* targeting of selected signaling pathways, we have developed an abbreviated five-stage protocol (25–30 days) to generate human Embryonic Stem Cell-Derived Beta-like Cells (ES-DBCs). We showed that Geltrex, as an extracellular matrix, could support the generation of ES-DBCs more efficiently than that of the previously described culture systems. The activation of FGF and Retinoic Acid along with the inhibition of BMP, SHH and TGF-beta led to the generation of 75% NKX6.1^+^/NGN3^+^ Endocrine Progenitors. The inhibition of Notch and tyrosine kinase receptor AXL, and the treatment with Exendin-4 and T3 in the final stage resulted in 35% mono-hormonal insulin positive cells, 1% insulin and glucagon positive cells and 30% insulin and NKX6.1 co-expressing cells. Functionally, ES-DBCs were responsive to high glucose in static incubation and perifusion studies, and could secrete insulin in response to successive glucose stimulations. Mitochondrial metabolic flux analyses using Seahorse demonstrated that the ES-DBCs could efficiently metabolize glucose and generate intracellular signals to trigger insulin secretion. In conclusion, targeting selected signaling pathways for 25–30 days was sufficient to generate ES-DBCs *in vitro*. The ability of ES-DBCs to secrete insulin in response to glucose renders them a promising model for the *in vitro* screening of drugs, small molecules or genes that may have potential to influence beta-cell function.

## Introduction

Type 1 Diabetes (T1D) is characterized by the autoimmune destruction of pancreatic beta-cells and the need for insulin therapy to control hyperglycemia. In some cases, pancreatic islet cell transplantation can reverse hyperglycemia in T1D and negate the use of insulin therapy [1]. Unfortunately, donor islet scarcity, ultimate graft failure and the required use of potentially harmful immune-suppressive drugs have restricted their use for the treatment of T1D [2]. Insulin-producing beta-like cells generated from embryonic stem (ES) cells or induced pluripotent stem (iPS) cells offer potential regenerative medicine approach that could be used instead of primary islet cell transplantation. To this end, laboratories have established multistep *in vitro* protocols to generate insulin-producing cells from ES and iPS cells. Although these differentiated cells have many features of *bona fide* human beta-cells, they fail to secrete insulin in response to *in vitro* glucose stimulation. In addition, significant percentages of the insulin-positive cells co-express other peptides including glucagon and somatostatin, which are not typically expressed in mature beta cells [3–6].

The differentiation of pluripotent stem cells (PSCs) to the Pancreatic Progenitor stage with subsequent kidney capsule transplantation has led to the generation of cells with a more beta-cell-like phenotype [7, 8]. Rezania *et al*. showed that these transplanted Pancreatic Progenitors could reverse hyperglycemia within 3–4 months in diabetic mice. This suggests that a population of cells within the preparation has the potential to develop into functioning beta-cells, if provided with the appropriate signals and growth factors in a temporally regulated manner [8]. Following this work, two groups have recently demonstrated that beta-like cell expansion protocols that include the inhibition of specific signaling pathways/molecules, can lead to the generation of highly glucose-responsive beta-like cells *in vitro* [9, 10]. Specifically, Rezania *et al*. reported that fully differentiated stage 7 ES-derived beta-like cells could lower blood glucose to normal levels in 6 weeks when transplanted into mice, while Pancreatic Progenitors could achieve this in 23 weeks [9]. Importantly, these cells were immature and contained clear deficiencies when compared to mature human islets [9]. Although this protocol could successfully generate 40% mono-hormonal insulin^+^/NKX6.1^+^ cells that express MAFA, it requires a long differentiation period (43 days) and a culture environment at the air-liquid interface; which may introduce many variabilities during long-term differentiation [9]. Pagliuca *et al*. [10] also established a method for the production of functional human beta-cells from ES cells via a three-dimensional cell culturing system. Using the same protocol, as *Pagliuca et al*, Millman *et al*. were able to differentiate human iPS cells derived from T1D patients into functional beta-like cells that were responsive to glucose challenges [11]. Although Pagliuca *et al*. showed an *in vitro* insulin secretion response of the ES-DBCs to glucose, they were unable to demonstrate an increase in MAFA expression which is required for the maturation and regulated secretion of insulin seen in mature beta-cells [10]. Despite these significant advancements, the differentiation protocols require *in vivo* maturation and/or extensive cell culture systems that are relatively costly.

Here, we describe a simple (five-stage) and shorter (25–30 days) protocol for the *in vitro* generation of ES-DBCs through Definitive Endoderm, Gut Tube Endoderm, Pancreatic Progenitors, Endocrine Progenitor and finally beta-like cell stages. This protocol uses Geltrex as a substrate to generate Definitive Endoderm and as a support for all of the differentiation stages throughout the protocol. As previously described by Russ *et al*., we similarly observed that inhibition of TGF-beta (ALK4, 5 and 7) and BMP signaling resulted in a high number of NGN3^+^/NKX6.1^+^ Endocrine Progenitors [12]. Further differentiation of the Endocrine Progenitors with combination of small molecules, including ALK5 inhibitor, thyroid hormone (T3), Notch and receptor AXL inhibitor, led to the generation of Insulin^+^/NKX6.1^+^/MAFA^+^ cells in a significant proportion of the differentiated cell population. Moreover, *in vitro* analyses of the ES-DBCs generated using this short protocol showed key features of human mature beta-cells and most notably their ability to sense and respond to changes in ambient glucose concentrations.

## Materials and Methods

### Cell culture

Human islets obtained from board-approved deceased donors were isolated by the Islet Core and Clinical Islet Laboratory at the University of Alberta, Canada. In all cases written consent from participants or their next-of-kin was obtained. Consent forms are kept in the Clinical Islet Laboratory at the University of Alberta. Use of the human islets in this study was reviewed and approved by University of Toronto Research Ethics Board (REB; Approval Number 20542). We used human H1 ES, human Epi-9 (an episomal reprogrammed iPS cell line) and iPS1-10 (an iPS cell line generated by doxycycline-inducible *PiggyBac*-expressing OCT4, SOX2, KLF-4 or c-Myc transposons as a monocistronic transcript established in Nagy laboratory) cells in this study. All PSCs were routinely cultured on mitotically inactivated Mouse Embryonic Fibroblast (MEF) feeder cells in hES medium: DMEM/F12 supplemented with 20% KnockOut Serum Replacement and 10 ng/ml bFGF (Invitrogen) and split at the ratio of 1:10–1:12 every 8–10 days using 100 ug/ml Collagenase type IV.

### In vitro differentiation of human PSCs

#### Stage 1: Definitive Endoderm (4 days)

All cells were cultured for three passages prior to the commencement of differentiation. To differentiate the PSCs into the Definitive Endoderm (DE) cells, H1 and iPS cells were dissociated using Accutase (STEMCELL Technologies) for 2 minutes. Next, H1 and iPS cells were re-plated onto Geltrex (0.1%, Invitrogen) coated 6-well plates in mTeSR feeder-free medium. To generate DE cells from PSCs, three different cell culture systems were tested; 1) culturing and differentiation on MEF, 2) culturing and differentiation on Geltrex (0.1%, Invitrogen) and, 3) Embryoid Body (EB) formation. For the first two cell culture conditions, differentiation started when the cells reached 60–70% confluency. To differentiate the cells as EBs, the dissociated single PSCs were subjected to EB formation in AggreWell^™^800 plates (STEMCELLS Technologies) for one day at a density of 1 x 10^6^ cells/ml in DMEM/F12 media supplemented with 3% KnockOut Serum Replacement. Next, 90–100 homogenously-shaped EBs were transferred to one well of a non-adherent 24-well plate where they underwent the differentiation procedure in suspension. To induce DE formation in all three-cell culture conditions, cells were treated with Activin A (100 ng/ml; R&D Systems) and Wnt3a (75 ng/ml; R&D System) in advanced-RPMI medium supplemented with 2% B27 and 1 mM sodium bicarbonate. This initial treatment with Activin A and Wnt3a is referred to as day 0 (D0) in the differentiation protocol. Over the next 3 days, the cells were induced using Activin A (100 ng/ml) in Advanced RPMI medium supplemented with 2% B27, 0.5 mM sodium bicarbonate and a 10 mM final glucose concentration. Media were replaced daily.

#### Stage 2: Gut Tube Endoderm (2 days)

To induce Gut Tube Endoderm formation from PSC-derived DE cells, the cells were induced by Keratinocyte Growth Factor (KGF; 50 ng/ml; R&D Systems) in Advanced RPMI medium supplemented with 2% FBS and 10 mM glucose.

#### Stage 3: Pancreatic Progenitor (4 days)

The differentiated cells from stage 2 were exposed to DMEM medium that was supplemented with 1% B27, KGF (50 ng/ml), KAAD-cyclopamine (25 μM), All-trans Retinoic Acid (2 μM), Noggin (100 ng/ml), ascorbic acid (VitC, 25mM) and 10 mM final glucose concentration for 4 days. The cell medium was changed every 2 days.

#### Stage 4: Endocrine Progenitor (6 days)

The cultures were continued for 3 days in DMEM medium supplemented with 1% B27, KGF (50 ng/ml), SB431542 (a TGF-beta receptors (ALK4, 5 and 7) inhibitor; final concentration 6 μM), Noggin (100 ng/ml) and 20 mM glucose. For the following 3 days, the cells were exposed to the same medium without KGF.

#### Stage 5: ES-Derived beta-like cells (9–14 days)

Differentiated cells from stage 4 were further differentiated using MCDB131 medium supplemented with 2% BSA, 100nM LDN193189 (a BMP receptor inhibitor), 1:200 ITS-X, 1 μM T3, 10 μM ALK5 inhibitor, 10 μM Zinc Sulfate, 100 nM gamma secretase inhibitor, Exendin-4 (50 ng/ml) and 20 mM glucose for the first two days, with the addition of 10 μg/ml of heparin for the subsequent three days. Next, the cells were exposed to MCDB131 medium further supplemented with 2% BSA, 1:200 ITS-X, 1 μM T3, 10 μM ALK5 inhibitor, 10 μM Zinc Sulfate, 1 mM N-acetyl cysteine, 10 mM Trolox (Vitamin E analogue), 2 μM R428 (receptor AXL inhibitor), 10 μg/ml of heparin, 50 ng/ml of Exendin-4, and 20 mM glucose for 5–7 days. To understand the effect of small inducers during stage 5, a group of differentiated cells from stage 4 was exposed to MCDB131 medium supplemented with 2% BSA and 20 mM glucose and cultured for 9–14 days only.

### Immunofluorescence staining

Human islets and the differentiated cells were fixed with 4% paraformaldehyde (PFA) for 30 minutes. After washing with PBS, cells were blocked and permeabilized with 5% BSA and 0.1% Saponin in PBS containing 0.1% TX-100 for 45 minutes at room temperature. They were then incubated with the corresponding primary antibodies listed in Table 1 for 2 hours or overnight. Next, the cells were washed three times with wash buffer (PBS without Ca^2+^ and Mg^2+^ containing 0.2% BSA, 0.1% TX-100 and 0.1% Saponin), and then incubated with the secondary antibodies for 45 minutes. The cells were washed 3 times and incubated with DAPI for 5 minutes for nuclei staining. The stained cells were visualized with a Leica (Houston, TX) TCS-SP2 confocal microscope.

**Table 1:** Antibodies information.

**Conjugated primary Ab**	**Company/Cat#**	**Application**	**Dilution**
PE-Mouse-CXCR-4	BD Cat# 561733	FC*	1:20
APC-Mouse-c-Kit	Thermo Fisher Cat# CD11705	FC	1:20
PE-Mouse-NKX6.1	BD Cat# 563023	FC	1:20
PE-Mouse-IgG2a isotype	BD Cat# 551438	FC	1:20
**Unconjugated primary Ab**	**Company/Cat#**	**Application**	**Dilution**
Rabbit FOXA2	Abcam Cat# ab40874	IF**	1:200
Goat SOX17	R&D Cat# AF1924	IF	1:200
Guinea Pig PDX1	Abcam Cat# ab47308	FC/IF	1:10000
Goat NGN3	Santa Cruz Cat # sc-13793	FC/IF	1:50
Rabbit NGN3	Abcam Cat# ab38548	FC/IF	1:100
Mouse C-peptide	Millipore Cat# 05–1109	FC/IF	1:100
Guinea Pig Insulin	Dako Cat# A0564	FC/IF	1:200
Mouse NKX6.1	Hybridoma Bank Cat# F55A12	IF	1:50
Rabbit MAFA	Custom Ab, Lifespan Biosciences, Seattle	IF	1:200
Rabbit Glucagon	Cell signaling Cat# D16G10	FC/IF	1:100
Rabbit Somatostatin	Thermo Fisher Cat# PA1-30636	FC/IF	1:100
Mouse NeuroD1	Abcam Cat# ab60704	IF	1:100
Mouse Syntaxin-1A	Thermo Fisher Cat# MA5-17612	IF	1:200
Rabbit Synaptophysin	Cell Signaling Cat# D35E4	IF	1:200
Rabbit ARX	Abcam Cat# ab111063	IF	1:25
Mouse PAX4	Hybridoma Bank Cat# M-Pax4-1F3A3	IF	1:25

* FC: Flow Cytometry

**IF: Immunofluorescent staining

### Flow Cytometry

For cell surface markers the differentiated cells from stage 1 were trypsinized using TrypLE 0.5X (Invitrogen) for three minutes and then centrifuged at 1000 RPM for 5 minutes. The cells were washed twice with FACS washing buffer (5% FBS in PBS without Ca^2+^ and Mg^2+^). Next, the cells were re-suspended in 90 μl of antibody dilution buffer (1% BSA in PBS without Ca^2+^ and Mg^2+^) and incubated with 5 μl of PE-conjugated CXCR-4 (CD184; BD Bioscience) and 5 μl of APC-conjugated c-Kit (CD117; Invitrogen) for 30 minutes on ice. Finally, the cells were washed three times with FACS washing buffer and analyzed using a Gallios^™^ Cytometer machine (Beckman Coulter).

For intracellular markers, the differentiated cells from stage 3, 4 and 5 were trypsinized with TrypLE 0.5X (Invitrogen) for 3 minutes and centrifuged at 1000 RPM for 5 minutes. After washing twice with the FACS washing buffer, the cells were fixed in 4% PFA for 10 minutes. After centrifugation, the cells were suspended in 100% methanol (Pre-chilled at -20°C) for 10 minutes at 4°C. After washing twice with FACS buffer, the cells were blocked with 10% FBS-containing PBS for 10 minutes at 4°C, and incubated for 2 hours or overnight with the corresponding primary antibodies (Table 1). Next, the cells were centrifuged and washed three times in the FACS washing buffer and blocked with 10% FBS-containing PBS for 10 minutes at 4°C prior to incubation with the secondary antibodies. Finally, the cells were washed 3 more times with FACS washing buffer and analyzed using a Gallios^™^ Cytometer machine (Beckman Coulter).

### Real time RT-PCR to quantify mRNA expression

Total RNAs were extracted from human islets, differentiated and undifferentiated cells using the RNeasy Mini Plus kit (Qiagen). The RNA (2–5 μg) was then reverse-transcribed using the TaqMan Reverse Transcription Kit (Applied Biosystems) and random hexamer primer mix according to the manufacturer’s instructions. For each reaction, the synthesized cDNA (20ng) was subjected to PCR by mixing with 5 μL of Power SYBR Green master mix (2X, Applied Biosystems), and 0.5 μM of each primer (Table 2) in a total volume of 10 μl. Precise pipetting was achieved using the automated pipetting epMotion 5075 workstation. The threshold cycle (Ct) of each target gene was normalized using the Ct of GAPDH as an internal standard. The comparative 2^-ΔΔCt^ method was applied to calculate the relative expression of target gene in each sample relative to the control. The relative gene expression values were presented as Mean±SEM of three independent biological experiments and three technical replicates.

**Table 2:** Primers information.

*Gene*	Accession #	Forward Sequence	Reverse Sequence
*ABCC8*	NM_001287174.1	GAGGCTACTTCACGTGGACC	CTATCTCGCTGTCAGGAAGGC
*Albumin*	NM_000477.5	GAAAAGTGGGCAGCAAATGT	GGTTCAGGACCACGGATAGA
*Amylase*	NM_000699.2	ACAATGATGCTACTCAGGTCAGA	TCGGCAATCTTAGAACGCAC
*ARX*	NM_139058.2	CCACGTTCACCAGCTACCAG	TCGGTCAAGTCCAGCCTCAT
*ATP5G3*	NM_001689.4	CGCATTGAGTCCCACTCCTT	ATATTGGGTGACAGGCGACG
*BHLHB3*	NM_030762.2	TAACCGCCTTAACCGAGCAA	GCGCATGTTTGAAATCCCGA
*Brachyury*	NM_001048.3	CAGGCGGGCAGCGAGAAG	AGGAAGGAGTACATGGCGTTGG
*BRN4*	NM_000307.4	GTCAAGGGCGTACTGGAGAC	TACAGAACCAGACACGCACC
*CACNA1A*	NM_000068.3	GTCGCCGTCATCATGGACAA	TGATACATGTCCAGGTAAGGCAT
*CACNA1D*	NM_000720.3	GGATCACCCAAGCTGAGGAC	CCACCAGCACCAGAGACTTC
*CGHA*	NM_001275.3	ACTGAAGGAGCTCCAAGACCT	GCCTCCTTGGAATCCTCTCTT
*CK19*	NM_002276.4	AGATGAGCAGGTCCGAGGTT	CAAGGCAGCTTTCATGCTCA
*EGR1*	NM_001964.2	CTTCAACCCTCAGGCGGACA	GAGTGGTTTGGCTGGGGTAA
*EPS1*	NM_001430.4	AACTTGTGCACCAAGGGTCA	CATGGAGAACACCACGTCA
*FOS*	NM_005252.3	GGGGCAAGGTGGAACAGTTA	AGGTTGGCAATCTCGGTCTG
*FOXA2*	NM_021784.4	AAGACCTACAGGCGCAGCT	CATCTTGTTGGGGCTCTGC
*GAPDH*	NM_001289745.1	CCTCAAGATCATCAGCAATG	CATCACGCCACAGTTTCC
*GATA4*	NM_002052.3	CTTGCAATGCGGAAAGAGGG	CTGACTGAGAACGTCTGGGAC
*GATA6*	NM_005257.5	AAGCGCGTGCCTTCATCA	TCATAGCAAGTGGTCTGGGC
*GCK*	NM_000162.3	CGGTCAGCAGCTGTATGAGA	TGTAGATCTGCTTGCGGTCG
*Glucagon*	NM_002054	GAATGAAGACAAACGCCACTCA	CGGCGGGAGTCGAGGTAT
*GLUT1*	NM_006516.2	GGCTTCTCCAACTGGACCTC	CCGGAAGCGATCTCATCGAA
*GLUT2*	NM_000340.1	GTCACTGGGACCCTGGTTTT	GTCATCCAGTGGAACACCCAA
*Gooscoid*	NM_173849.2	GCTTCTCAACCAGCTGCAC	CTGATGAGGACCGCTTCTG
*HCN3*	NM_020897.2	CTGGGCCTGAGCCTAAGAG	CAGCAGCATGATCAGGTCCC
*HEX*	NM_002729.4	AGCGAGAGACAGGTCAAAACC	TGGGCAAATCTTGCCTCTGAT
*HNF1B*	NM_000458.3	TCTCAACAAGGGCACCCCTA	GAAACAGCAGCTGATCCTGAC
*HNF4A*	NM_001287183.1	GGTGTTGACGATGGGCAATG	CTCGAGGCACCGTAGTGTTT
*HNF6*	NM_004498.2	TTAGCAGCATGCAAAAGGAAAGA	AGAGTTCGACGCTGGACATC
*HOPX*	NM_032495.5	GGTTTACCTCCTGCCCACG	CAGTGGGGCAGTCTGTCATT
*HPRT*	NM_000194.2	CCCTGGCGTCGTGATTAGTG	GCCTCCCATCTCCTTCATCA
*Insulin*	NM_001185097.1	AAGAGGCCATCAAGCAGGTC	TTCCCCGCACACTAGGTAGA
*ISL1*	NM_002202.2	ACGGTGGCTTACAGGCTAAC	ATTAGAGCCCGGTCCTCCTT
*KCNB1*	NM_004975.2	GAGTTCGATAACACGTGCTGC	TGGTGGAGAGGACGATGAAC
*KCNK1*	NM_002245.3	AGTCCTGGAGGATGACTGGAA	GCAATAAGGCCAAGTAGCAGG
*KCNK3*	NM_002246.2	CATCACCGTCATCACCACCA	CAGCAGGTACCTCACCAAGG
*KIR6*.*2*	NM_000525.3	GGACCCAGGTGGAGGTAAGG	CTCTCGGTGGGCACCTTCTC
*KLF9*	NM_001206.2	TACAGTGGCTGTGGGAAAGTC	CTCGTCTGAGCGGGAGAAC
*LZTS1*	NM_021020.3	AGCTCAGGTCCTACGAGAGG	CAGGATCTCGCTAGCCTTGG
*MAFA*	NM_201589.3	GAGAGCGAGAAGTGCCAACT	CTTGTACAGGTCCCGCTCTTT
*MAP2*	NM_002374.3	GCAGCTCTGCCTTTAGCAGC	TGCTTCTCTGACTCCTTTTCCT
*MESP1*	NM_018670.3	CCTGGTATCCGCCGTCCG	CATCCAGGTCTCCAACAGAGC
*MycN*	NM_001293228.1	CGCCCTAATCCTTTTGCAGC	TCCGCCCCGTTCGTTTTAAT
*NANOG*	NM_024865.2	ACCTCAGCTACAAACAGGTGAAG	TAAAGGCTGGGGTAGGTAGGT
*NeuroD1*	NM_002500.4	GAGGCCCCAGGGTTATGAGA	CCCACTCTCGCTGTACGATT
*NGN3*	NM_020999.3	CGCAATCGAATGCACAACCT	CTATGCGCAGCGTTTGAGTC
*NKX2*.*2*	NM_002509.3	CTTCTACGACAGCAGCGACA	TGTCATTGTCCGGTGACTCG
*NKX6*.*1*	NM_006168.2	TATTCGTTGGGGATGACAGAG	TGGCCATCTCGGCAGCGTG
*OCT4*	NM_002701.5	CAAAACCCGGAGGAGTCCCAG	CTCAAAGCGGCAGATGGTCG
*PAX4*	NM_006193.2	AGAAAGAGTTCCAGCGTGGG	CTTGGTACAGTCAGCCCCTG
*PCSK2*	NM_002594.4	TGCCGAAGCAAGTTACGACT	AACTTCTCCTGCACATCGGG
*PDX1*	NM_000209.3	AAGCTCACGCGTGGAAAGG	GGCCGTGAGATGTACTTGTTG
*PPY*	NM_002722.3	AATGCCACACCAGAGCAGAT	CGTAGGAGACAGAAGGTGGC
*PTF1a*	NM_178161.2	AGGCCCAGAAGGTCATCATC	TCCAGACTTTGGCTGTTCGG
*RCOR2*	NM_173587.3	CGAGGTCTTGACTCTCAGCTC	CATACCTACGGATGGCTTGAAC
*SLC30A8*	NM_001172815.1	CACTAGAAAGAAGGAGCTGCAA	TTTCCACTTGGCATAGGCGT
*Somatostatin*	NM_001048.3	ACGCAAAGCTGGCTGCAAGA	GGGGGCGAGGGATCAGAGGT
*SOX1*	NM_005986.2	CAACCAGGACCGGGTCAAAC	CCTCGGACATGACCTTCCAC
*SOX17*	NM_022454	ATGGTGTGGGCTAAGGAC	AGCGCCTTCCACGACTTG

### Digital Droplet RT-PCR (dd-RT-PCR)

For each dd-RT-PCR reaction mixture, the synthesized cDNA (50ng) was subjected to PCR by mixing with 12.5 μL of QX200 EvaGreen ddPCR supermix (2X, Bio-Rad) and 0.4 μM of forward and reverse primers (insulin primers listed in Table 2) in a total volume of 25 μl. Next, 20 μl of the dd-RT-PCR reaction mixture was loaded into the sample well and 70 μl of DG oil was loaded into the oil well of a DG8 cartridge. The cartridge was placed into the droplet generator to generate the oil-PCR reaction mixture. Then, 37.5 μl of the mix was loaded into each well of a 96-well PCR plate. The PCR was performed with an annealing temperature of 60°C for 40 cycles using a 2°C ramp rate. The positive and negative droplets were read on a QX200 droplet reader and the results were presented as exact insulin mRNA copy number per μl. GAPDH mRNA copy number was used as internal standard for the normalization.

### Glucose-stimulated insulin secretion (GSIS)

Differentiated ES-DBCs, EN cells at stage 5, or human Islets were subjected to a GSIS assay. The differentiated cells and human islets (~ 50 islets) were washed 2 times with KRB (Krebs-ringer bicarbonate pH 7.2; (112 mM NaCl, 4.8 mM KCl, 1.2 mM KH2PO4, 1.2 mM MgSO4, 2.5 mM CaCl2, 5 mM NaHCO3, 20 mM HEPES, and 0.1% BSA)) and then incubated with low glucose KRB for 60 minutes at 37°C. Next, the cells were incubated with KRB containing low glucose (2.8 mM), high glucose (16.5 mM), and both high glucose and KCl (16.5 mM glucose^+^30 mM KCL) for 30 minutes sequentially. To measure insulin and C-peptide content in the ES-DBCs at stage 5, the cells were suspended in Tris-EDTA (pH 7.4) on ice and then briefly sonicated until the cell membranes disappeared. The cell debris was removed via centrifugation and the intracellular insulin content was measured from the supernatant. Human insulin levels were measured using a Homogenous Time Resolved Fluorescence (HTRF) insulin assay kit (Cisbio), according to manufacturer’s instruction. C-peptide was measured using Ultra-Sensitive C-peptide ELISA kit (Mercodia), according to manufacturer instructions. All the Insulin and C-peptide measurements were performed on the PHERAstar FS (BMG Labtech). For normalization, the total protein contents of the cell lysates were measured using a Bradford assay.

### Dynamic insulin secretion by perifusion

Perifusion studies were done using stage 5 ES-DBCs alongside with human islets as a positive control. The differentiated ES-DBCs were cultured on a 6-well plate, dissociated using Accutase (STEMCELLS Technologies) for 5 minutes and then trypsinized (0.25% EDTA) for 5 minutes. 100 human islets or about 2×10^**6**^ ES-DBCs were washed twice with KRB buffer (112 mM NaCl, 4.8 mM KCl, 1.2 mM KH2PO4, 1.2 mM MgSO4, 2.5 mM CaCl2, 5 mM NaHCO3, 20 mM HEPES, and 0.1% BSA, pH 7.2) and pre-incubated for 60 minutes at 37°C in KRB buffer with low glucose. Next, the cells were centrifuged for 5 minutes at 1000 RPM and resuspended in 500 μl of Bio-gel P-4 (Biorep technologies) before being loaded into the plastic chambers of a PERI4.2 perifusion system with valve manifold (Biorep technologies). To dynamically stimulate the cells with glucose, they were perifused sequentially with 2.8 mM glucose containing KRB, 16.5 mM glucose containing KRB, 2.8 mM glucose containing KRB, 16.5 mM glucose containing KRB and lastly 16.5 mM glucose plus 30 mM KCl containing KRB. The cells were perifused at 100 μl per minute with KRB under temperature-controlled conditions and the supernatant from each cycle was collected for insulin measurement. After perifusion the ES-DBCs and islets were retrieved for protein measurement using a Bradford assay.

### Intracellular Ca2^+^ flux measurements

Cells were washed 3 times with imaging buffer (130 mM NaCl, 5mM KCl, 2mM CaCl2, 1 mM MgCl2, 5 mM NaHCO3, 10 mM HEPES) and incubated with Fluo-4, AM (1 μg/ml) in imaging buffer for 45 minutes at 37°C. The cells were then washed twice with imaging buffer. Intracellular Ca^2+^ was measured using a PHERAstar FS (BMG Labtech) by successive excitation of the Fluo-4, AM incubated cells. The emitted fluorescence signals were acquired at 480/500 nm and recorded as 20-second intervals per cycle (15 cycles for 2.8 mM glucose, 60 cycles for 16.5mM glucose, 15 cycles for 2.8 mM glucose, 60 cycles for 16.5mM glucose and 15 cycles for 16.5mM glucose and 30 mM KCl). The intracellular Ca^2+^ flux in each cell group was normalized to the intracellular Ca^2+^ flux measured in 2.8 mM glucose incubation as the baseline.

### Glucose metabolism

The respiration capacity of the non-treated, differentiated ES-DBCs compared to that of the MIN-6 beta-cell line was measured using mitochondrial flux kits with the Seahorse XF24 extracellular flux analyzer (Seahorse Bioscience, Billerica, MA, USA). To determine oxygen flux, the XF Cell Mito Stress Test Kit was used and Oxygen Consumption Rates (OCR) was measured according to the manufacturer's instructions. One hour before analysis, the culture medium of the cells was replaced with 525 μl of XF Base Medium (Seahorse Bioscience) containing 2mM glutamine, 1mM sodium pyruvate and 16.5 mM glucose, and the cells were incubated at 37°C without CO2. Four different components, XF Medium, Oligomycin (final concentration 5 μM), FCCP (final concentration 1 μM) and a cocktail of rotenone (5 μM) and Antimycin A (5 μM) were injected sequentially; three OCR measurements were taken after each injection. The results were normalized to baseline OCR.

### Statistical analysis

To analyze the real-time RT-PCR and seahorse data, an unpaired two-tailed *t*-test was used. For the statistical analyses of insulin and Ca^2+^ measurements, a paired one-tailed *t*-test was applied. The results in this study are presented as Means ± SD or Means ± SEM.

## Results

### Geltrex extracellular matrix induces efficient DE formation

**Fig 1:**
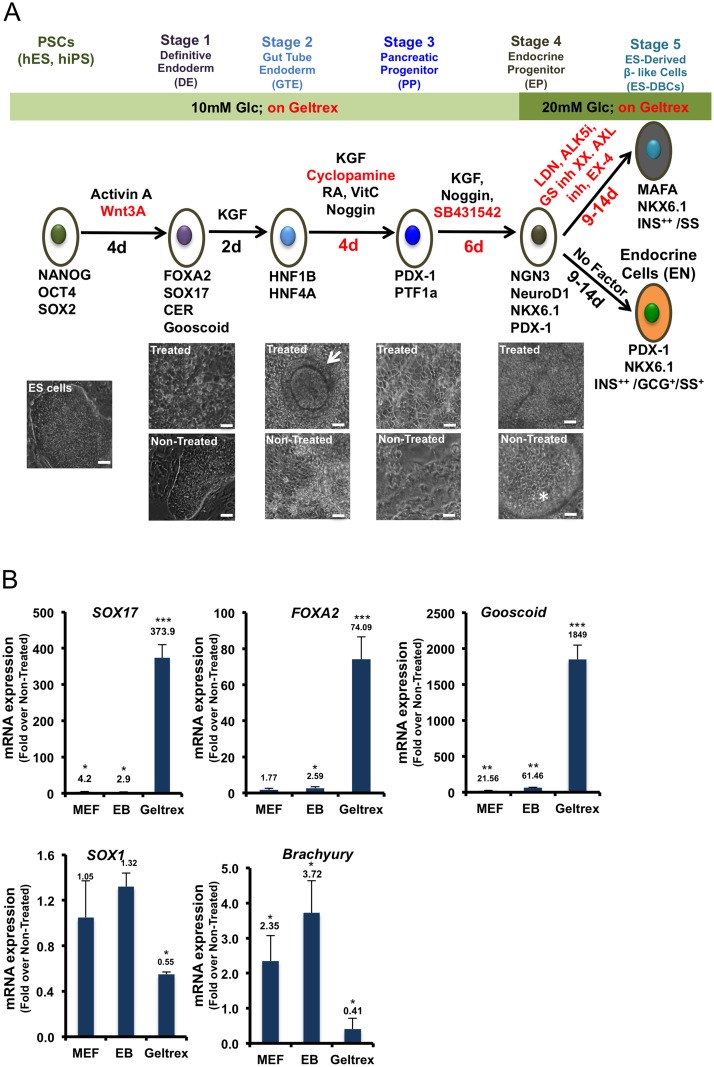
Short protocol outline. (A) Schematic overview of the 25 to 30-day protocol to generate human H1 ES-derived beta-like cells (DBCs). Below, images of the differentiated H1 cells and the control cells (Non-Treated ES cell) at each stage are shown. The arrow symbol identifies tube-like structure in the differentiated cells in the stage 2. The star symbol identifies detached dead cells as spheres in the Non-Treated cells in stage 4. Scale bar = 100μm for all cell images. The *red font* indicates modifications to molecules or timing in comparison to the protocol described by Rezania et al [9]. (B) Expression analyses of *SOX17*, *FOXA2* and *Gooscoid* as Definitive Endoderm (DE), *Sox1* as ectoderm, and *Brachyury* as mesoderm-specific markers in the H1 ES cells differentiated on MEF, Mouse Embryonic Fibroblast; as EB (Embryoid Bodies) or on Geltrex, analyzed by quantitative RT-PCR. (* *p< 0*.*05*, **p< 0.*01*, p***<*0*.*001*, significant differences between the treated and control cells in each condition, unpaired two-tailed *t*-test, n = 3).

The efficiency of DE formation during stage 1 determines the efficiency of the entire protocol leading to the generation of beta-like cells at the latter stages [13, 14]. To increase the consistency and efficiency of DE formation, we compared three different cell culture conditions. In the first condition, the cells were induced using Wnt3a/Activin A during embryonic body (EB) formation in suspension culture [13, 15]. In the second condition, the PSCs were plated on MEFs and induced with Wnt3a/Activin A [16]. To avoid any effects of undefined growth factors, such as BMPs and TGF-beta superfamily members that are produced by MEFs, in the third condition the cells were cultured on Geltrex extracellular matrix, a soluble form of reduced growth factor basement membrane extract, and were induced by Wnt3a and Activin A. The induction of the human H1 ES cells by Wnt3a and Activin A significantly increased expression of *SOX17*, *FOXA2* (not for MEF culture condition) and *Gooscoid* genes as specific markers of DE cells in all cell culture conditions. However, the levels of expression for all DE markers were significantly (*p<0*.*001*) higher in the cells cultured on Geltrex compared to the other conditions, as shown in Fig 1B. To analyze the effect of Geltrex on the derivation of non-endodermal cell layers, the expression levels of *SOX1* and *Brachyury* mRNAs as specific markers of neuroectoderm and mesoderm, respectively, were measured.

The results showed that the expression of *SOX1* and *Brachyury* (Fig 1B) was not up-regulated in the cells that were induced by Wnt3a/Activin A and cultured on Geltrex. These results imply that Geltrex did not induce mesodermal and ectodermal fates in the differentiated cells. The same DE-specific gene expression patterns were detected in Epi-9 and iPS1-10 cells that were differentiated on Geltrex: however, with less efficiency (Data not shown). Considering these results, the Geltrex extracellular matrix was used as a substrate for the differentiation of PSCs into pancreatic beta-like cells in our short protocol.

**Fig 2:**
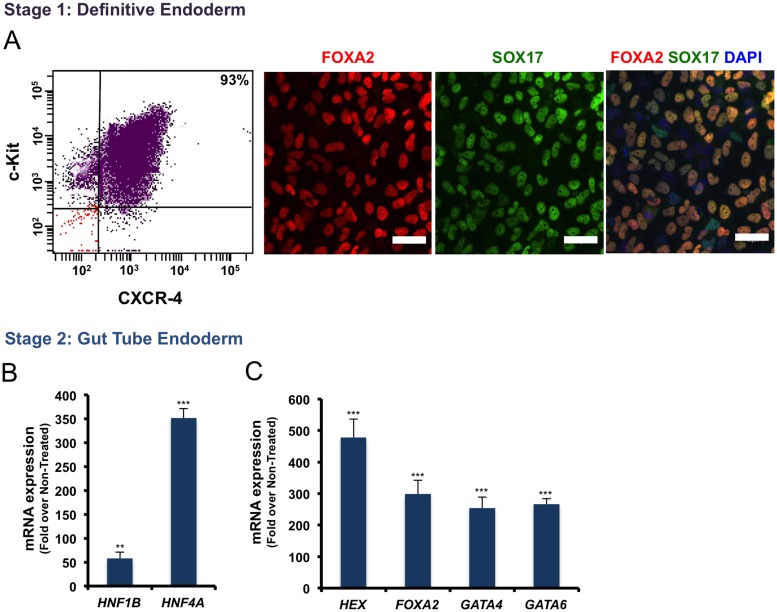
The efficiency of Definitive Endoderm (DE) and Gut Tube Endoderm formation at the stage 1 and 2 of the differentiation protocol. (A) Flow cytometry, and immunofluorescence staining for DE-specific markers in the differentiated H1 ES cells. (B) Quantitative RT-PCR results for Gut Tube Endoderm-specific markers are shown in (B), showing genes up-regulated in the stage 1, and (C) maintained highly expressed genes in the stage 2. Scale bar = 40μm. (*p< 0.*05*, **p< 0.*01*, p***<*0*.*001*, unpaired two-tailed *t*-test, n = 3).

Flow cytometry results (Fig 2A) showed that 93% of cells cultured on Geltrex and induced by Wnt3a/Activin A could express c-Kit (CD117) and CXCR-4 (CD184) as surface markers used for the quantification of DE formation efficiency in stage 1 [5]. Immunofluorescent staining for the DE specific markers, FOXA2 and SOX17 (Fig 2A), in the Wnt3a/Activin A treated ES cells showed that almost all of the cells co-expressed these markers in their nuclei. This implies that DE cells were generated with a high efficiency. We carefully checked flow cytometry to confirm DE formation efficiency in all the differentiation experiments. If the efficiency was lower than 90% (a minimum threshold), the experiment was terminated.

### Induction of the Pancreatic PDX1^+^ Progenitors

To generate Gut Tube Endoderm (GTE) we induced H1 ES-derived DE cells with KGF which is more potent than FGF10 [4, 7, 16], for 2 days (Fig 1A). The levels of *HNF1B* and *HNF4A* transcription factor mRNAs as markers of GTE cells were significantly increased (Fig 2B). As well the expression of *HEX*, *FOXA2*, *GATA4*, and *GATA6* (Fig 2C) transcription factors, which were up-regulated at stage 1, were maintained high as result of KGF induction. Additionally, tube-like structures were frequently observed in the KGF-treated cells (Fig 1A) but not in the non-treated cells (Fig 1A). In our protocol, we used RA in combination with KGF/FGF7, which is more effective than FGF10, to generate Pancreatic Progenitor cells [7]. In our short protocol we used Cyclopamine, and Noggin to inhibit SHH and BMP signaling pathways, respectively, as they are known to inhibit pancreas formation and PDX1 expression [17, 18]. We also tested the effect of PDBu (Phorbol 12, 13-dibutyrate; 100 nM) as a Protein Kinase C activator, and SANT-1 (0.25 μM) as a SHH inhibitor in our differentiation protocol. We found that the combination of VitC, RA, SANT-1 and/or PDBu is both acidic and cytotoxic for the differentiating cells, thus, KAAD-Cyclopamine was used instead of SANT-1 (data not shown).

**Fig 3:**
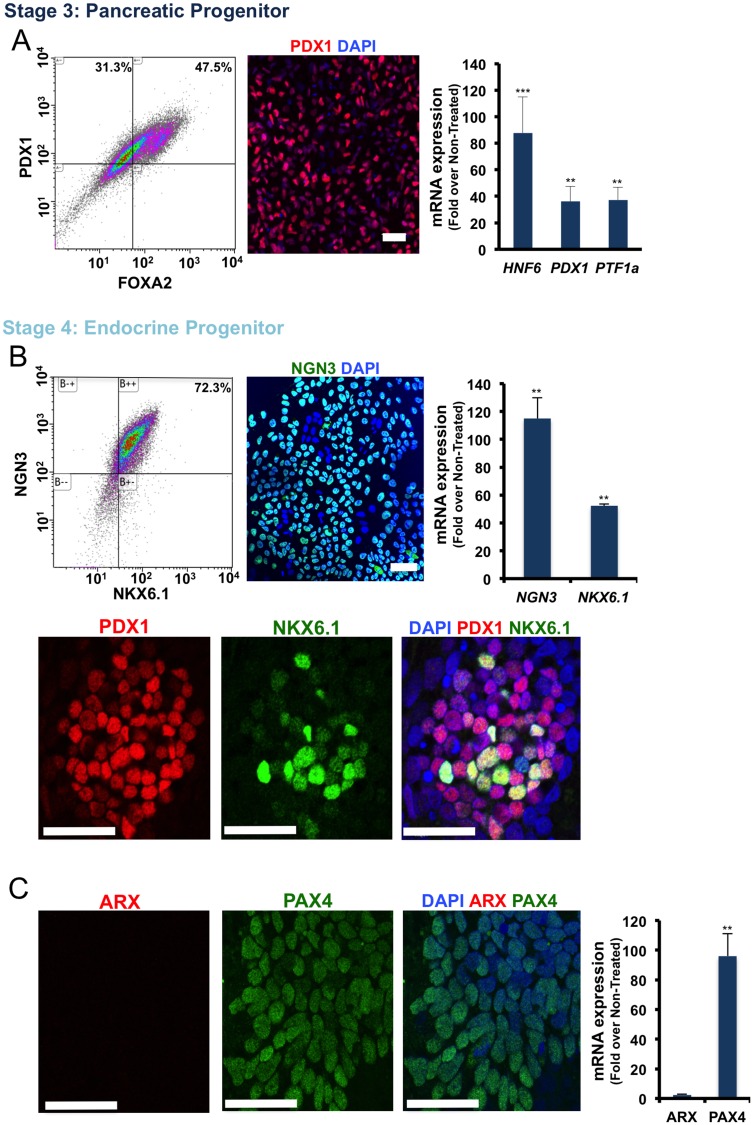
Characterization of the differentiated H1 ES cells at the Pancreatic Progenitor (PP) and the Endocrine Progenitor (EN) stages. (A) From left to right, flow cytometry for PDX1/FOXA2, immunofluorescence staining for PDX1, and qRT-PCR analysis for the PP-specific genes in the differentiated cells at stage 3. (B) Flow cytometry for NGN3/NKX6.1, immunofluorescence staining for NGN3, qRT-PCR analysis for the EP-specific genes and below, immunofluorescence staining for PDX1/NKX6.1 in the differentiated cells at stage 4. (C) Immunofluorescence staining for ARX/PAX4, and qRT-PCR analysis for ARX and PAX4 in differentiated cells at the stage 4. Scale bar = 40μm. (*p< 0.*05*, **p< 0.*01*, p***<*0*.*001*, unpaired two-tailed *t*-test, n = 3).

At stage 3, the differentiated cells exhibited an organized epithelial morphology in contrast to non-treated cells that assumed a mesenchymal-like morphology (Fig 1A). Flow cytometry results showed that more than 75% of the cells at stage 3 expressed PDX1 (Fig 3A). The immunofluorescence staining for PDX1 confirmed the flow cytometry results (Fig 3A). Transcript analysis of the stage 3 cells by real-time RT-PCR confirmed an increase in the levels of HNF6, *PDX1* and *PTF1a* expression in the ES-derived Pancreatic Progenitor cells (Fig 3A).

### Generation of NKX6.1^+^/NGN3^+^/NeuroD1^+^ Endocrine Progenitors

To further differentiate the ES-derived Pancreatic Progenitors into Endocrine Progenitors, stage 3 cells were induced with KGF for an additional 3 days. In addition, to continue the inhibition of BMP signaling, treatment with Noggin was extended into stage 4 for 6 days. Several studies have illustrated that the inhibition of TGF-beta receptors at stage 4 could efficiently increase the derivation of Endocrine Progenitors [4, 8, 9]. Here we used SB431542 to inhibit Activin receptor-like kinase [19] 4, 5 and 7. To generate Endocrine Progenitors (EN), the PSC-derived Pancreatic Progenitors were treated with a complex of KGF, SB431542 and Noggin in 10 mM glucose-containing medium for 3 days followed by further treatment with the same medium in the absence of KGF for an additional 3 days. Following this treatment, at the end of stage 4, 72–75% of cells were found to express NGN3/NKX6.1, as analyzed by flow cytometry (Fig 3B). Immunocytochemistry also confirmed expression of NGN3 in the nuclei of differentiated Endocrine Progenitor-like cells and the co-localization of NKX6.1 and PDX1 in the majority of the stage 4 cells (Fig 3B). Interestingly, the expression of NeuroD1 as a target of NGN3 [20] was observed in the differentiated stage 4 cells (S1 Fig). Quantitative RT-PCR also confirmed the flow cytometry and immunofluorescence staining results for NKX6.1 and NGN3 while showing high expression of *PAX4* in PSC-derived Endocrine Progenitor-like cells (Fig 3C). Immunofluorescent staining for PAX4 in the stage 4 cells confirmed a high number of PAX4-expressing cells in PSC-derived Endocrine Progenitor-like cells (Fig 3C). The study of transcription factors required for the generation of Endocrine Progenitor cells showed an increase in *FOXA2*, *HNF4*, *GATA4*, *ISL1 and NeuroD1* expression levels in the differentiated cells during stage 4 (S1 Fig). As shown in Fig 1A, cell death was observed in the non-treated cells during stage 4 whereas cell death and subsequent cell detachment in the differentiated Endocrine Progenitor-like cells was not observed (Fig 1A).

### Generation of Insulin-producing MAFA^+^/NKX6.1^+^Cells

To generate insulin-producing cells from Endocrine Progenitor-like cells, we employed two strategies. In the first strategy, the Endocrine Progenitor-like cells were differentiated without induction by exogenous factors for 9–14 days (Fig 1A) as previously described by Hrvatin *et al*. We referred to these differentiated cells as *ENdocrine cells* (EN). Our results showed that about 30% of differentiated EN cell populations were insulin^+^ cells, however, some of the cells were poly-hormonal and they expressed glucagon and/or somatostatin hormones in addition to insulin (data not shown). In the second strategy, PSC-derived Endocrine Progenitors were treated with LDN193189 (a BMP receptor inhibitor), ALK5 inhibitor, gamma secretase inhibitor XX (inhibitor of Notch signaling), receptor tyrosine kinase AXL inhibitor, T3 and Exendin-4 for 4–9 days (Fig 1A). We also used R428, an inhibitor of receptor AXL, to induce the expression of MAFA.

**Fig 4:**
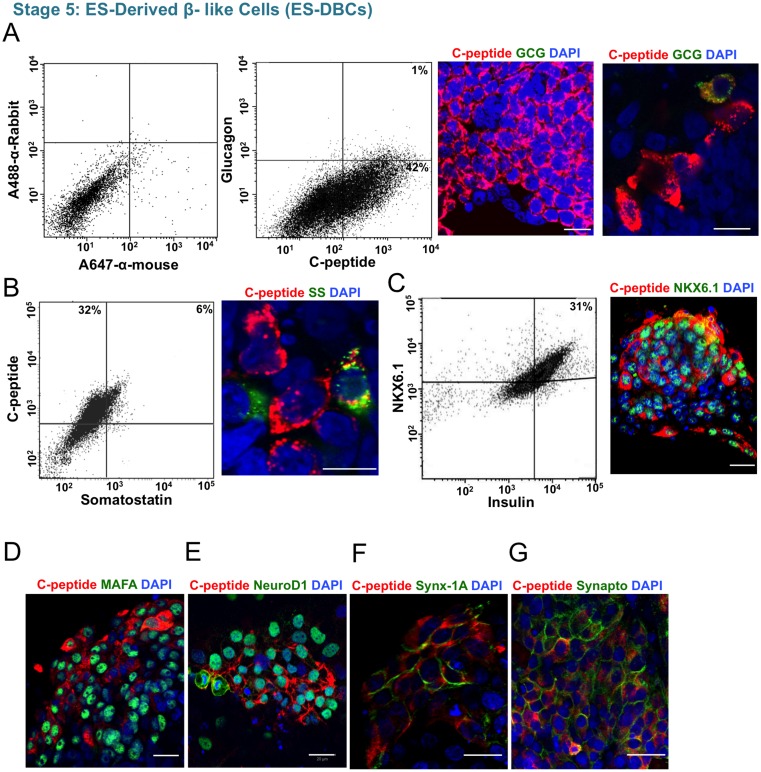
Study of insulin and beta-cell marker expression in the human H1 ES-DBCs at stage 5. (A) Flow cytometry and immunofluorescence staining for C-peptide/Glucagon. From left to right, gating of flow cytometry for detection of C-peptide and glucagon, flow cytometry for C-peptide and glucagon and immunofluorescence staining for C-peptide/glucagon in the ES-DBCs. (B) Flow cytometry and immunofluorescence staining for C-peptide/Somatostatin and (C) Insulin/NKX6.1, in the ES-DBCs. (D) Immunofluorescence staining for C-peptide/MAFA, (E) C-peptide/NeuroD1,(F) C-peptide/Syntaxin-1A, and (G) C-peptide/Synaptophysin in the ES-DBCs at the stage 5. Scale bar = 20μm. GCG: Glucagon, SS: Somatostatin.

Flow cytometry results showed that 35–40% of the differentiated ES-Derived Beta-Like Cells (ES-DBCs) could synthesize insulin *de novo*, as we analyzed C-peptide expression (Fig 4A). Less than 1% of the C-peptide^**+**^ ES-DBCs also co-expressed glucagon (Fig 4A), and about 6% of the cells co-expressed C-peptide and somatostatin (Fig 4B). Flow cytometry analysis using antibodies against insulin and NKX6.1 as markers of mature and functional beta-cells, showed that 30% of the cells express both proteins (Fig 4C). We also detected MAFA expression in the C-peptide expressing cells (Fig 4D). NeuroD1 as a target of NGN3 was also expressed in the ES-DBCs (Fig 4E). The expression of syntaxin-1A as a key protein in synaptic exocytosis (Fig 4F), and Synaptophysin as an endocrine marker (Fig 4G) were detected in the membrane of some C-peptide-expressing ES-DBCs [21].

**Fig 5:**
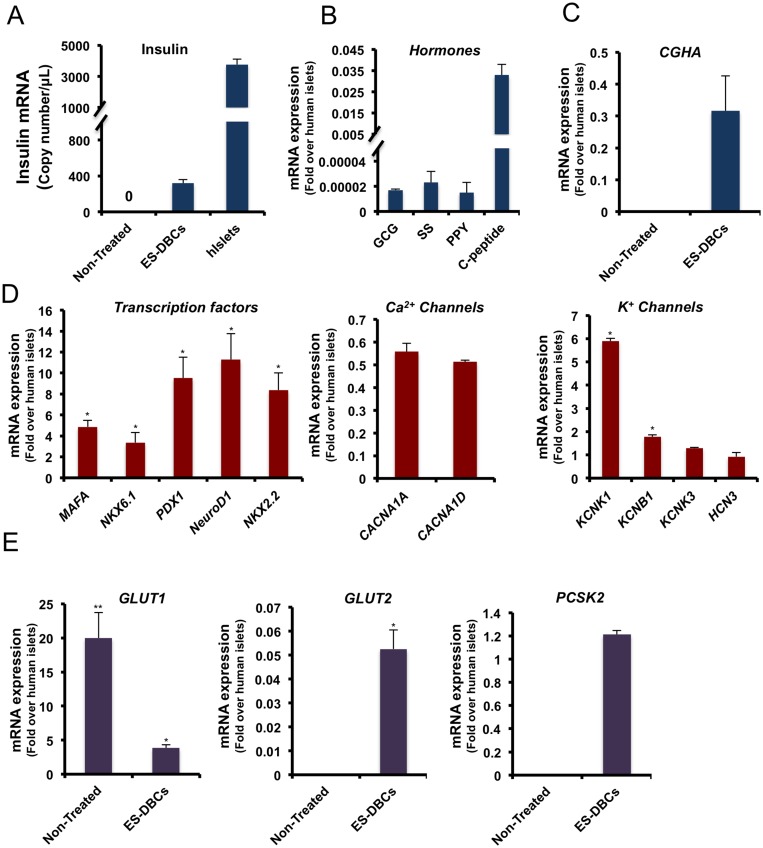
The mRNA expression analysis of pancreatic islet, beta-cell and related genes in the differentiated human H1 ES-DBCs. (A) Exact copy number of insulin mRNA molecules in the ES-DBCs and human islets by digital droplet RT-PCR (GAPDH was used for normalization). Quantitative real time RT-PCR analysis for (B) endocrine hormones, (C) Chromogranin A, (D) pancreatic transcription factors, Ca^+2^ and K^+^ channels genes, (E) Glucose transporters (*GLUT1 and 2*) and *PCSK2* as the enzyme required for pro-insulin processing and in the ES-DBCs compared to human islets. (*p< 0.*05*, **p< 0.*01*, p***<*0*.*001*, unpaired two-tailed *t*-test, n = 3).

Our results showed that although human EPi-9 and iPS1-10 as iPS cell lines could differentiate into insulin-producing cells through the protocol, the efficiency was significantly reduced compared to H1 ES cell lines. Digital droplet RT-PCR (dd-RT-PCR) results demonstrated that ES-DBCs expressed 319 insulin mRNA copies per microliter of the PCR reaction (399 mRNA molecules/ 20 ng of RNA), whereas H1 ES and non-treated cells expressed no insulin mRNA copies (Fig 5A). The copy number of insulin mRNA for human islets was 3763 copies per microliter of the PCR reaction (4703 mRNA molecules/ 20 ng of RNA). Some batch-to-batch and donor-to-donor variation was observed in both ES-DBCs and primary human islet cells. These variations are not unexpected for both human islets and ES-DBCs generated through a 25–30 day protocol involving four basal media and 20 differentially combined factors (Fig 1A).

As shown in Fig 5B, the expression analyses of other hormones in ES-DBCs indicate very low expression of glucagon (GCG; 1.7×10^−5^), somatostatin (SS; 23×10^−5^) and pancreatic polypeptide (PPY; 15×10^−5^). ES-DBCs could express a high level of the transcription factors *PDX1*, *NKX6*.*1 NeuroD1*, *NKX2*.*2*, *MAFA*, and Chromogranin A (*CGHA*) as a marker of endocrine cells (Fig 5C). Several glucose-sensing genes were also found to be elevated in ES-DBCs as shown in Table 3. To test the specificity of our short protocol for the generation of beta-like cells specifically, we analyzed the expression of other cell linage specific markers in the ES-DBCs at the end of stage 5. As shown in Table 3, quantitative expression of non-beta-cell lineage markers including Amylase (marker of acinar cells), CK19 (marker of ductal cells), Albumin (marker of hepatic cells), MAP2 (marker of neurons), and (E and F) OCT4 and Nanog (markers of pluripotent stem cells), were not increased in the ES-DBCs.

**Table 3:** Gene expression analysis of human H1 ES-DBCs. The data are presented as fold changes over Non-Treated human H1 ES cells.

Gene	Expression (Folds)	Function/Marker
*KIR6*.*2*	10.53	KATP channel
*ABCC8*	8.5	KATP channel
*SLC30A8*	3.35	Zinc transporter
*GCK*	8.62	Glucokinase
*ATP5G3*	9.59	ATP synthase
*Amylase*	0.83	Acinar
*CK19*	0.58	Ductal
*Albumin*	No expression	Hepatic
*MAP2*	0.43	Neurons
*OCT4*	0.06	Pluripotency
NANOG	0.52	Pluripotency

One of the issues related to the generation of beta-like cells from PSCs is the presence of poly-hormonal cells among the differentiated cells. Following the sequential inhibition of signaling pathways throughout our differentiation protocol, flow cytometry illustrated that less than 1% of the ES-DBCs express insulin and glucagon together (Fig 4A) and 6% express insulin and somatostatin together (Fig 4B). To understand why such a small number of α-cells were detected at the end of stage 5, we investigated the expression of TFs governing the commitment of α-cell precursors to mature α-cells during stage 4. As shown in Fig 3C, the expression of *ARX*, a transcription factor involved in α-cell development [22, 23], was not significantly (*p>0*.*05)* up-regulated at stage 4 compared to non-treated cells. Immunofluorescent staining for ARX revealed no positive cells within the differentiated Endocrine Progenitors at stage 4, whereas PAX4, which is indispensable for the development of beta and δ cells [24, 25], was abundantly expressed in mRNA and protein levels.

### Comparing gene expression of ES-DBCs with fetal and adult beta-cells

**Fig 6:**
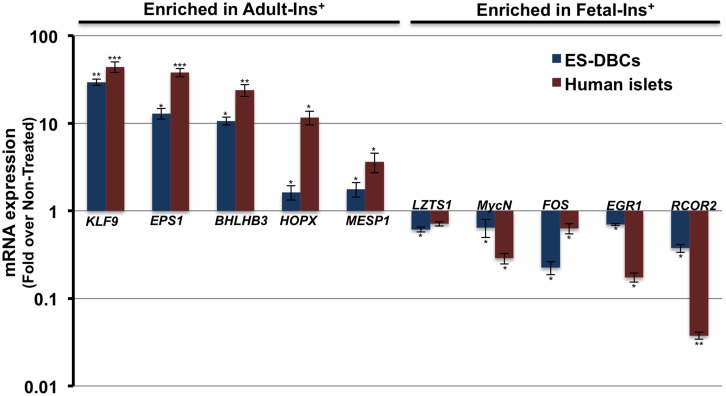
Comparison of gene expression in human H1 ES-DBCs and mature beta-cells. Expression of the top-ten most significantly enriched mRNAs in either adult mature or fetal beta-cells as described by *Hrvatin et al. [26]* were examined in ES-DBCs vs. the human adult islets via real time RT-PCR assay. (*p< 0.*05*, **p< 0.*01*, p***<*0*.*001*, unpaired two-tailed *t*-test, n = 3).

Recently, Melton’s group reported the transcriptome profiles of fetal and adult human insulin positive beta-cells [3, 26]. To comprehend the maturity of the differentiated ES-DBCs at the transcriptome level, from this study, we selected the 5 the most enriched genes in fetal insulin positive beta-cells and 5 from adult insulin positive beta-cells to examine their expression in our cells. We show that LZTS1 (Leucine Zipper, putative Tumor Suppressor 1), MycN (N-Myc), FOS (FBJ murine osteosarcoma viral oncogene), EGR1 (early growth response 1) and RCOR2 (REST co-repressor 2) which were enriched in fetal sorted beta-cells, were down-regulated in ES-DBCs compared to non-treated cells (Fig 6). In contrast, the expression of KLF9 (Kruppel-like factor 9), EPS1 (Endothelial PAS domain protein 1), BHLHB3 (basic helix-loop-helix, E41), HOPX (HOP homeobox) and MESP1 (mesoderm posterior bHLH transcription factor 1) which were enriched in the adult sorted insulin positive beta-cells, were up-regulated in the differentiated ES-DBCs (Fig 6). These results suggest that the pattern of gene expression in differentiated ES-DBCs is closer to adult mature beta-cells than fetal beta-cells, at least in terms of the top-ten modulated genes from the human fetal or adult sorted insulin-positive beta-cells.

### De novo insulin synthesis and secretion in differentiated ES-DBCs at the stage 5

**Fig 7:**
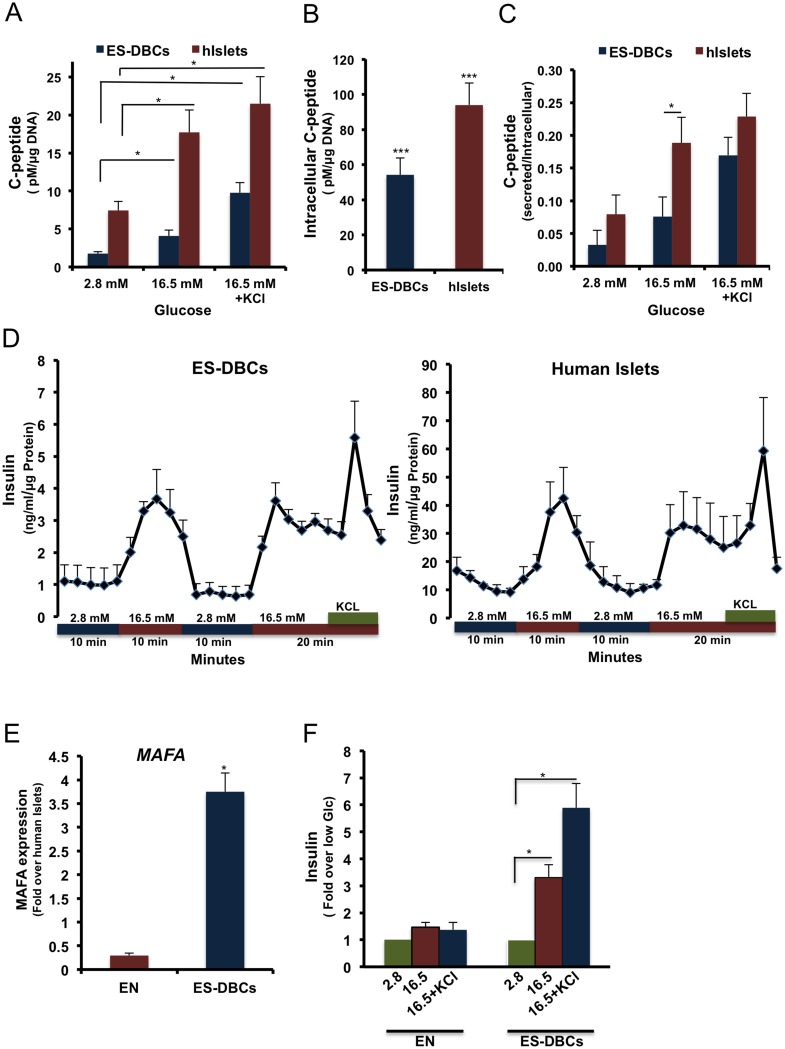
Examination of beta-cell stimulus-secretion coupling in human ES-DBCs vs. human islets. (A) Measurement of C-peptide in the supernatant, and (B) lysates of H1 ES-DBCs and the human islets after stimulation by glucose. (C) Normalized secretion compared to intracellular C-peptide. (D) Temporal insulin secretion by perifusion in ES-DBCs and human islets. Correlation between (E) MAFA expression analyzed by qRT-PCR and (F) insulin secretion, in response to glucose stimulation in EN and ES-DBCs at stage 5. EN: ENdocrine cells as referred in Fig 1A. (*p< 0.*05*, **p< 0.*01*, p***<*0*.*001*, paired two-tailed *t*-test, n = 5).

One of the critical issues regarding the generation of insulin-producing cells from stem cells is the ability of the differentiated cells to sense changes in glucose concentrations and secrete insulin accordingly. We performed glucose-stimulated insulin secretion assays in both static and dynamic assays. Glucose-challenged ES-DBCs secreted 3-fold more insulin in response to high glucose compared to low glucose concentrations (Fig 7F), whereas the endocrine cells (EN) that were spontaneously differentiated at stage 5 were unable to secrete insulin in response to glucose (Fig 7F).

The human C-peptide ELISA showed 2.8 and 5.2 fold (*p*< 0.05) increases in response to 16.5 mM glucose and 16.5 mM glucose containing 30 mM KCl KRB buffer, respectively, compared to the low glucose condition (Fig 7A). As shown in Fig 7B, the intracellular content of insulin in the ES-DBCs that were differentiated through the short protocol was 54.1 pM/μg DNA, whereas the spontaneously differentiated non-treated cells contained 1.2 pM/μg DNA of intracellular insulin. Furthermore, we compared the amount of secreted C-peptide in the differentiated ES-DBCs stimulated with glucose to the secreted C-peptide in the human islets (Fig 7C). The secreted C-peptide in ES-DBCs was increased from 1.8 pM/μg DNA in the low glucose treatment to 4.1pM/μg DNA in the high glucose treatment and finally to 9.1 pM/μg DNA in the high glucose plus KCl treatment. Although both the ES-DBCs and isolated human islets showed a regulated glucose-stimulated insulin secretion pattern, the amount of secreted C-peptide in human islets was greater in both the low and high glucose challenge conditions. Next, we graphed the ratio of secreted C-peptide to intracellular C-peptide content in both ES-DBCs and human islets. In the high glucose treatment, the ratio for human islets was approximately 2 times greater (*p*< 0.05) than the ratio in ES-DBCs (Fig 7C); however it was not statistically different between the ES-DBCs and the human islets in low and high glucose under depolarizing conditions (KCl) (Fig 7C).

To further analyze the physiological glucose response of ES-DBCs, we performed islet perifusion studies to better mimic physiological conditions (i.e. 2 rounds of sequential low/high glucose challenges). As shown in Fig 7D, the ES-DBCs at the end of stage 5 could respond to the dynamic glucose stimulation in the first and second rounds of high glucose challenge. This suggested that the ES-DBCs could repeatedly secrete insulin in response to high glucose stimulation like isolated human islets (Fig 7D); however the amount of secreted insulin in the human islets was remarkably higher.

### MAFA expression and glucose responsiveness of the ES-DBCs

To increase the expression and nuclear localization of MAFA, a crucial transcription factor involved in the maturity of ES-DBCs, we treated the differentiating cells with R428, N-acetyl cysteine and Trolox during stage 5 (Fig 1A). Next, GSIS assays were carried out and the same cells were subjected to real time RT-PCR quantification to measure MAFA expression. The results illustrated that treatment of the cells with MAFA-inducing factors could increase the level of MAFA mRNAs (Fig 7E; about 4-fold more than the human islets), allowing the ES-DBCs to secrete 3 fold more insulin in response to the glucose stimulation compared to the low glucose condition (Fig 7F). Conversely, in the ENdocrine cells (EN cells) that were not treated with MAFA-inducing factors, the level of MAFA expression was 3.5-fold lower than human islets (Fig 7E). Interestingly, EN cells were not responsive to glucose stimulation (Fig 7F).

### Calcium flux and mitochondrial dynamics to assess glucose sensing

**Fig 8:**
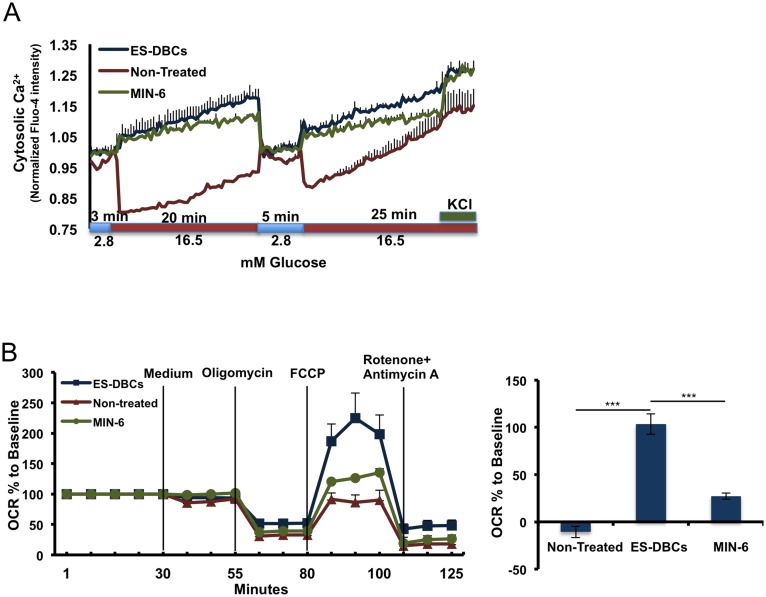
Analyses of Ca^+2^ flux, and respiration capacities of the human H1 ES-DBCs. (A) Measurement of glucose-stimulated cytosolic Ca^+2^ flux in the ES-DBCs, Non-Treated cell and MIN-6 beta-cell population. (B) Mitochondrial respiration (the potential of mitochondria to reserve energy) in ES-DBCs, Non-Treated and MIN-6 cells using the seahorse technique. (n = 4)-two technical replicates per batch, data are presented as Mean±SD. (*p< 0.*05*, **p< 0.*01*, p***<*0*.*001*, paired two-tailed *t*-test, n = 4).

To further characterize our ES-DBCs, the intracellular Ca^2+^ flux that occurs in response to glucose stimulation was measured. As depicted in Fig 8A, ES-DBCs and MIN-6 cells (control) responded to sequential glucose stimulation by repeatedly increasing intracellular Ca^2+^ in a pattern that was consistent with glucose responsiveness. Interestingly, spontaneously differentiated non-treated cells showed a pattern of Ca^2+^ flux in response to glucose challenges that was opposite to ES-DBCs (Fig 8A).

Mitochondrial respiration capacity during stage 5 was examined by measuring mitochondrial respiration. To measure mitochondrial stress in the ES-DBCs we used 1μM FCCP, an uncoupler to short-circuit the proton path and permit maximal respiration as measured by OCR (Oxygen Consumption Rate), in the third injection. Next, a cocktail of rotenone (5 μM) and Antimycin A (5 μM) was injected to inhibit electron transfer and attenuate the OCR. The difference between maximum and basal respiration shows the spare capacity, referring to the potential of mitochondria to reserve energy during acute aerobic stress. As shown in Fig 8B, the OCR measurement demonstrated that the ES-DBCs had the greatest maximal respiration and spare capacity, illustrating that the mitochondria in these cells have more energy reserves available to handle the demands of acute stress when compared to the other cell types.

## Discussion

The ability to generate beta-like cells from human pluripotent stem cells *in vitro*, would provide a unique tool for screening novel therapies that target beta-cells and to speed the development of cell replacement therapies for Type 1 diabetes. Recently, two groups developed remarkably similar protocols for generation of so-called glucose-responsive ES-DBCs *in vitro*, which could reverse hyperglycemia in diabetic mouse models [9, 10]. Although, the ES-DBCs generated in both studies possessed several molecular and physiological characteristics of natural human islets, the researchers reported that they still displayed some characteristics of immature beta-cells [9, 10]. For example, Rezania *et al*. reported that the differentiated cells at stage 7 have a delayed insulin secretion and Ca^2+^ influx in response to glucose [9] while Pagliuca *et al*. did not demonstrate the expression of mature beta-cell markers, such as MAFA in the ES-DBCs [10]. Moreover, the Pagliuca protocol is performed in 500ml spinner flasks and requires 5 different media and a myriad of growth factors. This makes the protocol expensive for adaptation to smaller scale screening of drugs, genes and bioactive molecules that may be involved in beta-cell function [10]. Although the Rezania protocol can be utilized on a smaller scale, it is temporally demanding (43 days) and requires an air-liquid interface for culturing. Here, we have established a five-stage protocol that is short (25–30 days) where all steps are performed *in vitro* without the requirement of a complicated cell culture system.

In our report, we demonstrated that Geltrex as an extracellular matrix, could better support DE formation compared to other cell culture systems. We also determined that DE formation is the most important checkpoint in our protocol, as the experimental batches that contained a DE cell number below the threshold at stage 1, could not differentiate into glucose-responsive cells at stage 5. Furthermore, we found that the induction of cells with KGF, RA, Cyclopamine (SHH inhibitor) and Noggin (BMP inhibitor) at stage 3, results in 80% PDX1^+^ Pancreatic Progenitor cells. Rezania *et al*. recently described that the treatment of the PSC-derived differentiated cells at an early stage of differentiation with ascorbic acid (VitC) would reduce the early expression of *NGN3*. NGN3 is master regulator of pancreatic endocrine cell differentiation, which is thought to promote the generation of poly-hormonal cells [9]. In our protocol, we did not observe significant differences in the *NGN3* expression after adding VitC to the cells at stage 2. Unlike the Rezania *et al*. protocol that used MCDB 131 culture medium with no VitC, we used Advanced RPMI that contains VitC and found that increasing VitC concentration did not appear to have any additional effect. Several studies have shown that signaling molecules secreted by surrounding mesenchymal tissue at E12.5 such as FGF-10 and RA could promote the generation of pancreatic PDX1^+^ progenitors in the developing pancreas [27]. We also tested combinations of inducers described by the Rezania and Pagliuca protocols at stage 3 and found them to be more toxic than our components, perhaps due to the differences in the cell culture and differentiation systems.

Inhibition of TGF-beta (ALK4, 5 and 7) using SB431542, and the BMP4 signaling pathway at stage 4 resulted into 70% NKX6.1^+^/NGN3^+^ Endocrine Progenitors. To generate mono-hormonal insulin^+^/NKX6.1^+^ cells, the Endocrine Progenitors were treated with thyroid hormone T3, and gamma secretase inhibitor XX, as inhibitor of Notch signaling. It has been proposed that growth arrest specific protein 6 (GAS6), an agonist of the AXL receptor tyrosine kinase subfamily, plays a role in beta-cell maturation through the down-regulation of Mafa expression in rodents [28]. Thus, to increase the expression of MAFA, which is indispensable for the maturation of ES-DBCs [9], we treated the cells with R428 as a receptor AXL inhibitor [28].

The results showed that at the end of stage 5, about 35% of differentiated cells were mono-hormonal insulin^+^ and only 1% and 6% were insulin^+^/glucagon^+^ and insulin^+^/somatostatin^+^, respectively. One explanation could be the low expression of ARX, a transcription factor that is needed for α-cell development at stage 4. Additionally, 30% of the insulin^+^ cells co-expressed NKX6.1, which is expressed in glucose responding cells [29].

Perifusion studies showed that ES-DBCs could respond to repeated stimulations including glucose challenges. However, insulin secretion was lower in absolute magnitude relative to the human islets. A potential reason is lower intracellular insulin content in the ES-DBCs compared to human islets (Fig 7B). The amount of intracellular C-peptide in the ES-DBCs was found to be about half of the human islets and the ratio of secreted C-peptide to intracellular insulin content in the ES-DBCs during the high glucose condition, was about two fold less than the ratio for human islets. This could potentially indicate the presence of immature beta-like cells in the ES-DBC population. Like Rezania *et al*., our results also confirmed that a high level of MAFA expression is a prerequisite for regulated glucose-stimulated insulin secretion.

Glucose-stimulated insulin secretion is tied to glucose metabolism and subsequent calcium influx. It is also known that calcium influx essentially mirrors insulin secretion and is a requisite signaling molecule to trigger insulin exocytosis [10]. We found that the pattern of Ca^2+^ flux in our ES-DBCs and in response to glucose challenge was in line with the pattern shown by the Melton group in stem-cell-derived beta-cells [10]. Consequently, a clear correlation between glucose-stimulated increased Ca^2+^ influx and insulin secretion was established. To assess glucose metabolism in our ES-DBCs, respiration capacity was assessed using the Seahorse platform. The ability to metabolize glucose and stimulated insulin secretion is consistent with a more mature beta-like cell than what we had previously reported [30]. Furthermore, Melton’s group recently profiled the transcriptome of fetal immature and adult mature insulin^+^ cells sorted by insulin antibody followed by RNA-Seq analysis [3, 26]. Upon comparison with their results, the pattern of gene expression in our ES-DBCs appear to be more similar to adult mature beta-cells than fetal/immature beta-cells.

In conclusion, we have developed an abbreviated and simplified *in vitro* protocol for the generation of glucose-responsive, ES-derived beta-like cells. The majority of the insulin-producing cells were mono-hormonal and demonstrated many key characteristics of mature beta-cells. We believe that this protocol could be applied to platforms for screening drugs, small molecules, and genes that may improve beta-cell function.

## Supporting Information

S1 Fig Expression analysis of Endocrine Progenitor-related transcription factors in the human H1 ES-derived Endocrine progenitor cells. (A-E) Quantitative RT-PCR analyses of *FOXA2*, *HNF4*, *GATA4*, *ISL1* and *NeuroD1* transcription factors in the differentiated Endocrine Progenitors cells. (F) Immunofluorescence staining for NuroD1 in the ES-derived Endocrine progenitors. (*p< 0.05, **p< 0.01, p***<0.001, paired two tailed t-test, n = 3).
(TIF)

## Abbreviations

ES-DBCs: Embryonic Stem Cell-Derived Beta-like Cells
iPS cells: induced Pluripotent Stem cells
PSCs: Pluripotent Stem Cells
DE: Definitive Endoderm
T1D: Type 1 Diabetes
