# Supplementary figures and images for: An Abbreviated Protocol for In Vitro Generation of Functional Human Embryonic Stem Cell-Derived Beta-Like Cells
Source: PLoS One. 2016 Oct 18;11(10):e0164457. doi: 10.1371/journal.pone.0164457 (PMC5068782; doi:10.1371/journal.pone.0164457)

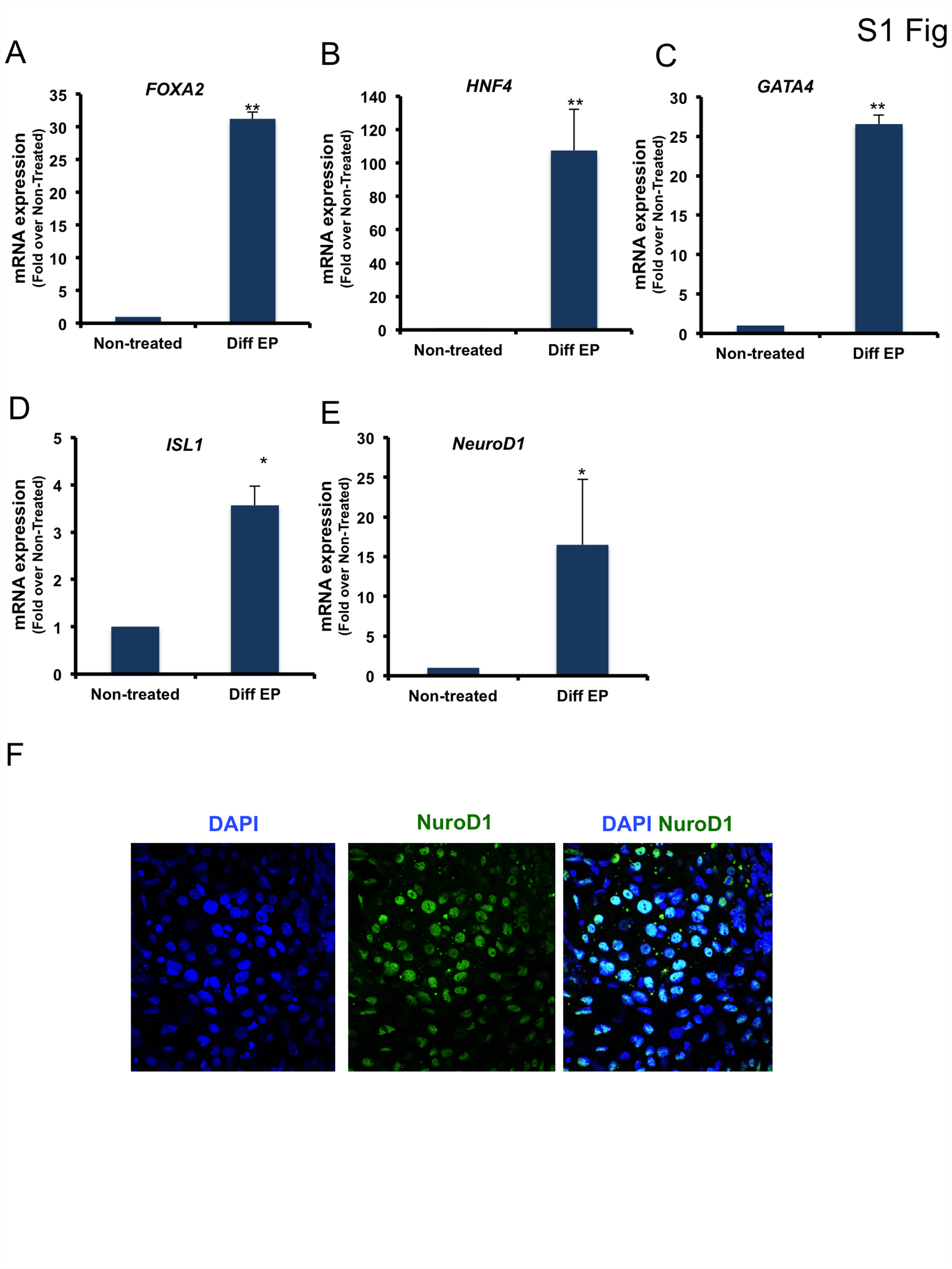

Supplement: S1 Fig — (A-E) Quantitative RT-PCR analyses of FOXA2, HNF4, GATA4, ISL1 and NeuroD1 transcription factors in the differentiated Endocrine Progenitors cells. (F) Immunofluorescence staining for NuroD1 in the ES-derived Endocrine progenitors. (*p< 0.05, **p< 0.01, p***<0.001, paired two tailed t-test, n = 3). (TIF) [file pone.0164457.s001.tif]
